# Characterization of exercise-induced hemolysis in endurance horses

**DOI:** 10.3389/fvets.2023.1115776

**Published:** 2023-04-27

**Authors:** Patrycja D. Pakula, Anna Halama, Eman K. Al-Dous, Sarah J. Johnson, Silvio A. Filho, Karsten Suhre, Tatiana Vinardell

**Affiliations:** ^1^Equine Veterinary Medical Center, Qatar Foundation, Doha, Qatar; ^2^Department of Physiology and Biophysics, Weill Cornell Medicine—Qatar, Doha, Qatar; ^3^Department of Endurance Racing, Al Shaqab, Doha, Qatar; ^4^College of Health and Life Sciences, Hamad Bin Khalifa University, Qatar Foundation, Doha, Qatar

**Keywords:** equine, endurance, hemolysis, exercise-induced, metabolomics (OMICS)

## Abstract

Exercise-induced hemolysis occurs as the result of intense physical exercise and is caused by metabolic and mechanical factors including repeated muscle contractions leading to capillary vessels compression, vasoconstriction of internal organs and foot strike among others. We hypothesized that exercise-induced hemolysis occurred in endurance racehorses and its severity was associated with the intensity of exercise. To provide further insight into the hemolysis of endurance horses, the aim of the study was to deployed a strategy for small molecules (metabolites) profiling, beyond standard molecular methods. The study included 47 Arabian endurance horses competing for either 80, 100, or 120 km distances. Blood plasma was collected before and after the competition and analyzed macroscopically, by ELISA and non-targeted metabolomics with liquid chromatography–mass spectrometry. A significant increase in all hemolysis parameters was observed after the race, and an association was found between the measured parameters, average speed, and distance completed. Levels of hemolysis markers were highest in horses eliminated for metabolic reasons in comparison to finishers and horses eliminated for lameness (gait abnormality), which may suggest a connection between the intensity of exercise, metabolic challenges, and hemolysis. Utilization of omics methods alongside conventional methods revealed a broader insight into the exercise-induced hemolysis process by displaying, apart from commonly measured hemoglobin and haptoglobin, levels of hemoglobin degradation metabolites. Obtained results emphasized the importance of respecting horse limitations in regard to speed and distance which, if underestimated, may lead to severe damages.

## 1. Introduction

Hemolysis is defined as the process involved in the breakdown of erythrocytes, characterized by impairment and disruption of the cell membrane leading to the release of hemoglobin (Hb) to the bloodstream (1–3). Hb binds and carries oxygen obtained from the lungs to supply muscles and other organs, it also possesses the ability to eliminate carbon dioxide, the waste product of its metabolism (4, 5). After the rupture of the erythrocytes, Hb is further broken down into globin and heme—the particle containing iron. Heme is then converted into biliverdin, and eventually into bilirubin, and excreted in the bile (6, 7). Free plasma Hb and its metabolites have deleterious effects on the cells, and numerous molecules are engaged into plasma clearance, such as haptoglobin (Hp) (8, 9). Heme is scavenged by albumins, hemopexin, and low-density lipoproteins, which prevent its toxic effect (6). They also contribute to iron recycling and limit pathogens access to heme iron that is contained in enzymes and regulatory proteins essential for the bacteria physiological maintenance (10, 11).

Hemolysis may be caused by numerous factors, including immune-mediated reactions, infectious agents, hemoparasites, chemical substances, plants, as well as hypo-osmolality, hypophosphatemia, or hepatic insufficiency (8, 12, 13). Exercise-induced hemolysis is a pathophysiological process observed in endurance exercise, well described in human runners, and caused by mechanical and/or metabolic factors. Among the mechanical factors, foot strike is considered the most significant. When the foot hits the ground during a running process, the foot capillaries are compressed, leading to mechanical damage and rupture of erythrocytes (14). This type of hemolysis appears not only during running activity, but also in sports disciplines such as endurance swimming, rowing, or cycling, in which foot strike does not occur. Hemolysis in those athletes is induced by constant, repeated muscle contractions that leads to capillary vessels compression, contributing to the injury and rupture of erythrocytes (15). Vasoconstrictions of internal organs, especially kidneys, due to the increased blood flow demand in skeletal muscles, also contributes to the injury and rupture of the erythrocytes (16). Multiple metabolic factors can cause an increased fragility of erythrocytes and thus hemolysis. This includes metabolic acidosis caused by high level of lactates, increased body temperature, and increased catecholamines level, as well as osmotic and oxidative stress (17–23).

Although hemolysis is considered a physiological phenomenon, it may become a severe pathological hurdle (24). Increased levels of Hb lead to free-radicals generation, with high reactivity and result in cellular and tissue damage (8, 25). Hb also decreases nitric oxide (NO) bioavailability, critical for smooth muscle tone regulation, platelet activation, and aggregation modulation (26, 27). Therefore, due to NO scavenging by free Hb, hemolysis leads to smooth muscle dystonia that can result in gastrointestinal contractions, dysphagia, abdominal pain, vascular constriction, hypertension, and intravascular thrombosis (28). Moreover, Hb and heme possess pro-inflammatory properties (29). Erythrocytes have been shown to be a source of at least 45 cytokines, both pro- and anti-inflammatory, suggesting that hemolysis potentially contribute to the inflammation process due to release of those cell signaling molecules (30, 31).

Exercise-induced hemolysis is reported in equine athletes in various disciplines (32, 33). Hemolysis has been observed in standardbred horses, where the concentration of plasma Hb increased and plasma Hp decreased after a race, in comparison to the values before the race (33). A similar trend was described in a study conducted on thoroughbred horses during training, where different hemolysis patterns were linked to equine gender (32). Moreover, the association between exercise and increased osmotic fragility of the erythrocytes (OFE), known to be one of the factors contributing to hemolysis, was described in show jumping horses (34). Another interesting study on Thoroughbred horses exercising on the treadmill, suggested that the OFE could be linked with a decreased pH and increased temperature of the blood (35).

One of the most challenging disciplines for the equine metabolism is endurance racing, where the horse is expected to run distances of up to 160 km. The number of eliminated horses in endurance racing oscillate around 50% (36) and the most common causes of elimination are orthopedic problems and metabolic reasons (36). The latest include dehydration, electrolytes loses, lactic acid production, and heat generation (37, 38). This fact was corroborated by a French study where 12.5% of the competing endurance horses required treatment for metabolic conditions (39). Murakami and collaborators showed that exercise-induced hemolysis occurred in horses running for 2.2 km for 5 consecutive days displaying an increase in plasma Hb correlated with the severity of the exercise (40).

The most popular methods to detect hemolysis are the measurement of cell-free Hb and Hp in blood plasma with conventional, widely accessible assays (1, 41). Nowadays, metabolomics can provide a comprehensive view of the whole set of molecules within the cell/tissue, by using high-throughput techniques that generate large amounts of information, which are further analyzed with bioinformatics. These advanced techniques quantify peptides, fatty acids, amino-acids, carbohydrates, and many others, enabling the understanding of the interactions and dependencies between them (42, 43). Metabolomics was used in our previous study on endurance competing horses. It provided an in-depth characterization of metabolic alterations induced during the races and we identified potential metabolic predictors of performance. In this study, a significant increase in heme, biliverdin, and bilirubin as well as a correlation between plasma color change, indicative of hemolysis, and average speed was noted (38).

Overall, none of the previous described studies have studied exercise-induced hemolysis in horses combining conventional methods with OMICS sciences and performed performance correlations with the level of molecules involved in hemolysis. Therefore, the aim of this study is to provide an in-depth characterization of the exercise-induced hemolysis in endurance horses in order to better understand this process and prevent its detrimental consequences.

## 2. Materials and methods

### 2.1. Experimental group

The study was conducted on horses participating in three different endurance race events at the Qatar Endurance Village in Mesaieed, Qatar. Forty-seven horses eligible to enter the race according to FEI and/or Qatar Endurance Committee were included. No discrimination of age, breed, or sex was implemented. The group included 6 stallions, 19 geldings, and 22 mares, all of them Arabians or half-breed Arabians. The mean age was 12 ± 3.3 years old. The study was approved by the Institutional Animal Care and Use Committee of Weill Cornell Medicine, Qatar, under the number WCMQ-2018-003 and owner consent was obtained.

### 2.2. Study design

All horses passed an initial veterinary health check. Each race was divided into loops, of 20 to 40 km long. After each loop, horses underwent a veterinary health check and compulsory rest time of at least 1 min for each kilometer ran. Based on the veterinary evaluation, horses were either eliminated or qualified to continue the race. The number of loops completed by the horse were noted in order to determine the actual distance ran, as the animal could have been eliminated thus not completing the distance registered for the race (40, 60, 80, 100, or 120 km). In this study, samples were obtained from horses competing in races of 80, 100, and 120 km. Heart rate and blood samples were collected from each horse before the race (BR), specifically during the afternoon in the day preceding the race, and after finishing or being eliminated from the race (AR; within 30 min). The reasons of elimination included metabolic problems, lameness, and trainer/rider withdrawal. The number of horses sampled per race event was 13, 23, and 26, respectively. Thirty-four horses were sampled in one race, 11 horses were sampled during two races, and 2 horses were sampled in 3 races, giving 62 sets of samples. Each set contained samples from before and after the race, giving total number of 124 samples.

### 2.3. Sample collection and macroscopic plasma assessment

Blood was collected from the jugular vein centrifuged at the race premises and plasma was aliquoted, transported and stored for further analysis at −80°C at the Equine Veterinary Medical Center. BR and AR samples from each horse underwent macroscopic plasma color assessment and grading. Four grades of plasma color change were distinguished: 0 = no or minimal change, 1 = mild change, 2 = moderate change, and 3 = severe color change. A total of 59 pairs of plasma samples were assessed and each horse obtained its own score.

### 2.4. Osmolality measurements and ELISA

Osmolality was measured at the Anti-Doping Lab in Qatar (ADLQ), using a freezing-point Fiske Micro-Osmometer Model 210 (Norwood, MA, United States). Osmolality was measured in duplicate, using a 20 μL of plasma for each sample. Enzyme-linked immunosorbent assay (ELISA) was utilized to measure the levels of cell-free hemoglobin and haptoglobin. ELISA test was performed on all samples in duplicate with the Horse Haptoglobin ELISA Kit and Horse Hemoglobin ELISA Kit (CD Creative Diagnostics®, NY, United States). Tests were performed according to the commercial protocol, absorbance was measured at 450 nm in a Microplate Reader Infinite M Nano Tecan© (Männedorf, Switzerland), and the concentration was calculated with GraphPad Prism Software© [Version 8.4.3 (686), 2020, San Diego, CA, United States]. In total, results from 116 samples for Hp and 115 samples for Hb levels were obtained.

### 2.5. Metabolomic measurement

Metabolic measurements were conducted at ADLQ by deploying implemented Metabolon Inc. HD4 platform as previously described (34). Briefly, the samples extracts, obtained from methanol-based solvent extraction with recovery standards, were divided into equal parts and evaporated under nitrogen stream [TurboVap (Zymark)]. The samples were further reconstituted in four different solvents compatible with each analytical methods as previously described (38). The metabolic measurements were conducted with Waters ACQUITY ultra-performance liquid chromatography (UPLC) and a Thermo Scientific Q-Exactive high resolution/accurate mass spectrometer interfaced with a heated electrospray ionization (HESI-II) source and Orbitrap mass analyzer operated at 35,000 mass resolution (44). The raw data were analyzed at Metabolon Inc. (Durham, NC, United States) using Metabolon’s hardware and software for compound identification. The obtained peaks were compared to library entries of purified, and the components were identified based on standard retention index, their accurate mass match to the library ±10 ppm, and MS/MS forward and reverse scores (45). Each obtained compound was corrected in a run day and all the metabolic data were normalized to correct for variations resulting from inter-day tuning differences in the instrument.

### 2.6. Statistical analysis

Statistical analysis was performed using GraphPad Prism Software© [Version 8.4.3 (686), 2020, San Diego, CA, United States]. The results obtained were tested using paired *t*-test, Wilcoxon test, as well as one-way ANOVA and Kruskal–Wallis test. Correlation between variables were computed with Pearson and nonparametric Spearman correlation tests. The data normality was tested using Anderson-Darling, D’Agostino and Pearson, Shapiro–Wilk, and Kolmogorov–Smirnov tests. Significancy threshold was set at *p* ≤ 0.05. Data were expressed as mean ± SEM.

## 3. Results

### 3.1. Hemolysis parameters before and after the race

**Figure 1:**
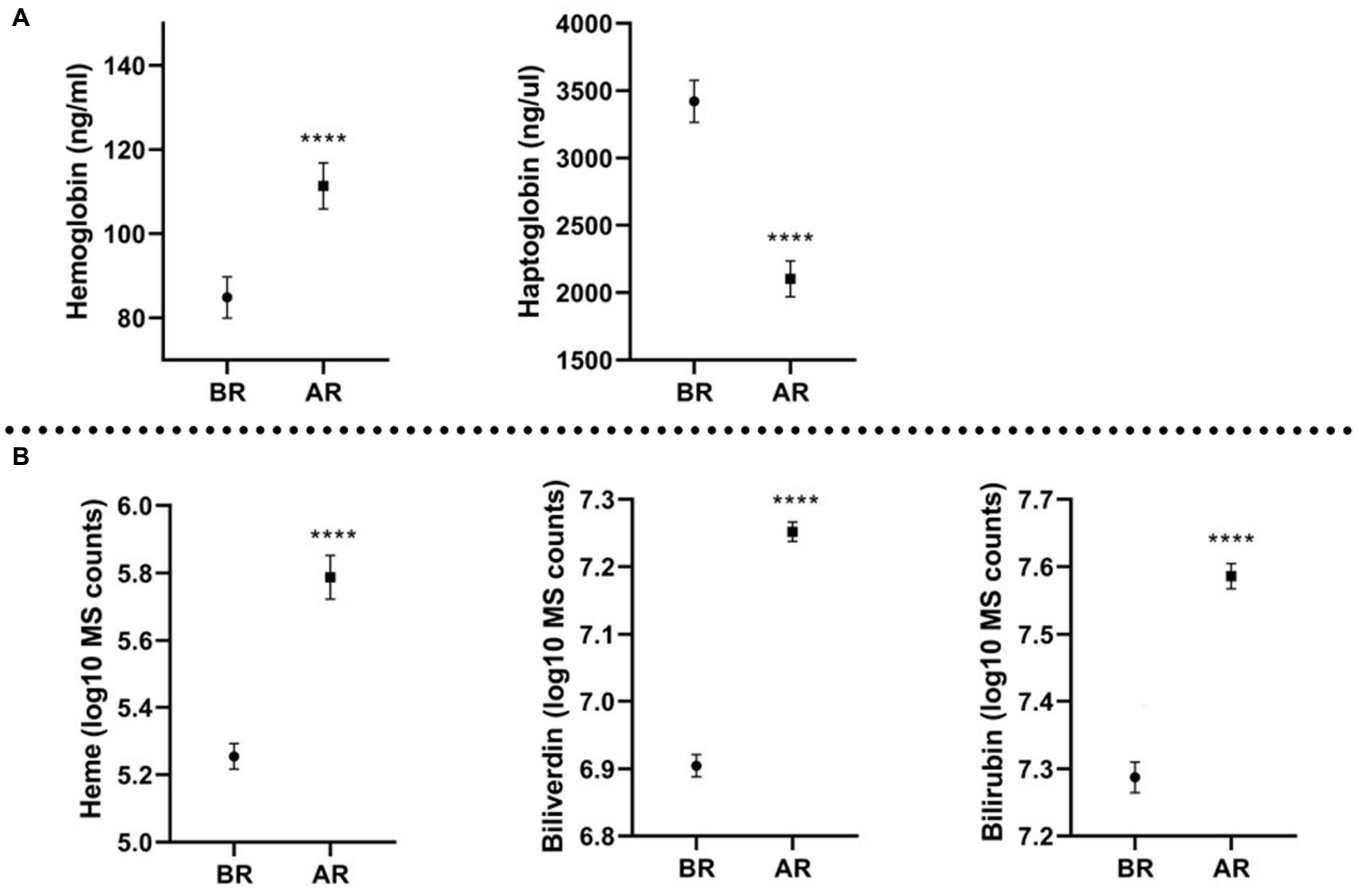
Molecules quantification (mean and standard error of the mean) before the race (BR) and after the race (AR); **(A)** molecules measured with ELISA, expressed in ng/mL (Hb) and ng/μL (Hp); **(B)** molecules measured with metabolomics platform, expressed in log10 MS counts; ^****^p ≤ 0.0001.

Hb, heme, bilirubin, and biliverdin were significantly increased after the race (*p* ≤ 0.0001) and Hp was significantly decreased after the race (*p* ≤ 0.0001). Levels of molecules before and after the race are displayed on Figure 1. Mean values and Standard Error, as well as fold change, are shown in Table 1. Moreover, the majority of the samples displayed a change in plasma color; orange or reddish plasma samples after the race compared to the yellow samples collected before the race, this suggests rupture of red blood cells. The above color change is in agreement with identified increase in the Hb and other hemolysis parameters.

**Table 1:** Mean levels and standard error of the mean for hemolysis molecules studied before the race (BR), after the race (AR) and their fold change.

Molecule	Unit	Before the race (mean ± SEM)	After the race (mean ± SEM)	*p*-Value	Fold change
Hemoglobin (ELISA)	ng/μL	84.88 ± 4.88	111.40 ± 5.44	<0.0001	1.40
Haptoglobin (ELISA)	ng/mL	3422.00 ± 157.00	2103.00 ± 133.40	<0.0001	0.65
Heme	log10 MS counts	5.26 ± 0.038	5.79 ± 0.065	<0.0001	6.83
Biliverdin	log10 MS counts	6.91 ± 0.016	7.25 ± 0.014	<0.0001	2.30
Bilirubin	log10 MS counts	7.29 ± 0.023	7.59 ± 0.019	<0.0001	2.07

*p*-values express the statistical difference between BR and AR.

### 3.2. Hemolysis parameters and elimination reason

**Figure 2:**
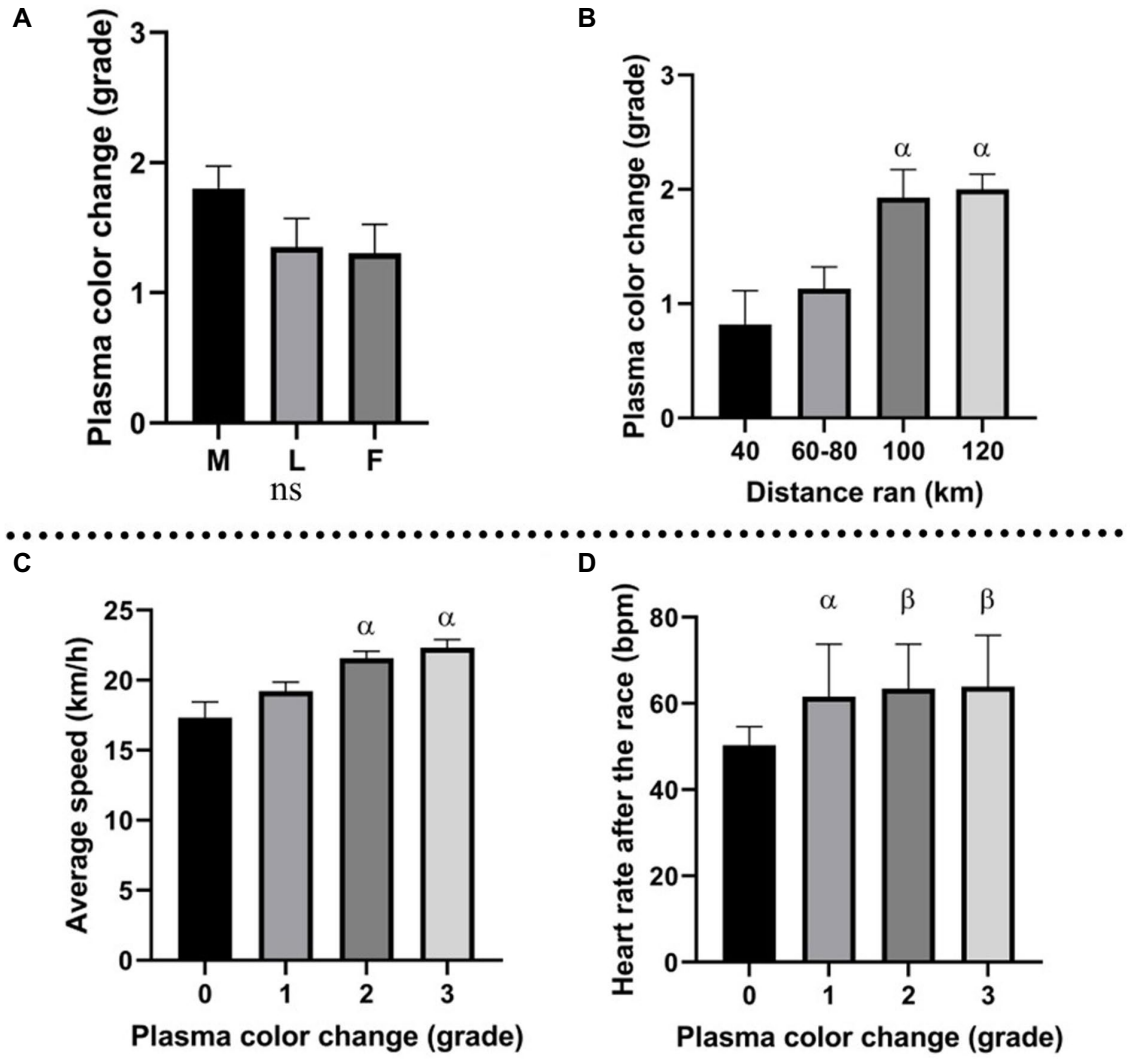
**(A)** Plasma color change (grade) in horses eliminated for metabolic reasons (M), lameness (L), and horses that finished the race (F); **(B)** association between distance ran by the horses and plasma color change grade; α: *p* ≤ 0.05 vs. 40 km; **(C)** association between plasma color change grade and average speed during the race; α: *p* ≤ 0.001 vs. grade 0; **(D)** association between plasma color change (grade) and HR after the race (bmp); α: *p* ≤ 0.05, vs. grade 0, β: *p* ≤ 0.01 vs. grade 0.

**Figure 3:**
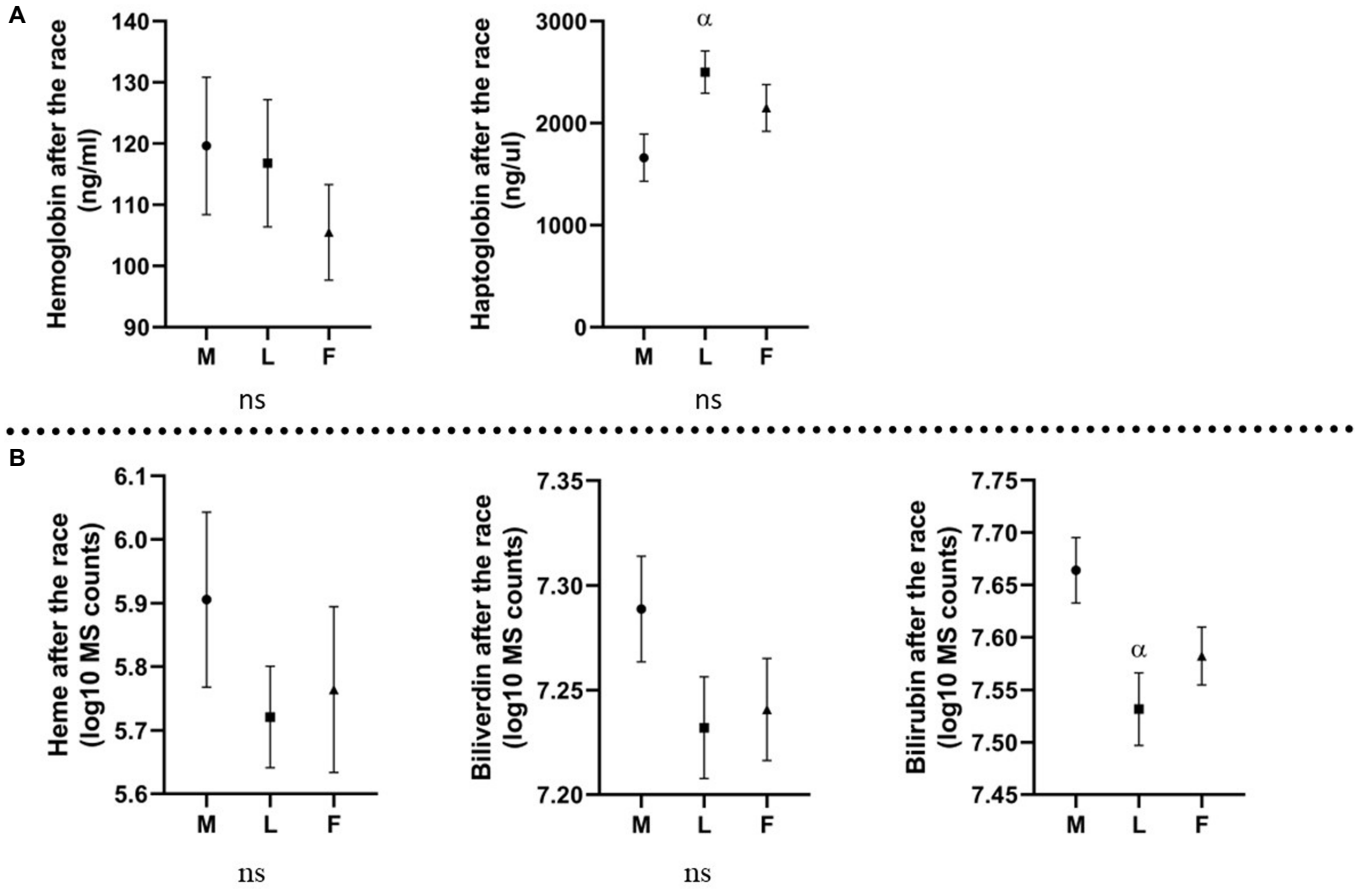
Levels of molecules (Mean and standard error of the mean) in horses eliminated for different reasons. M, metabolic eliminations; L, lameness eliminations; F, finishers; **(A)** molecules analyzed with ELISA, expressed in ng/mL (Hb) and ng/μL (Hp); **(B)** molecules analyzed with metabolomics platform, expressed in log10 MS counts; α: *p* ≤ 0.05 vs. metabolic eliminations.

All horses were divided into three groups based on the race outcome: horses eliminated for metabolic reasons, horses eliminated for lameness, and horses that finished the race. Plasma color change (average grade) was the highest in the group eliminated for metabolic reasons (Figure 2A). In this group, the concentration of Hb was the highest, and Hp the lowest. Moreover, all analyzed metabolites (heme, biliverdin, and bilirubin) displayed the highest levels in metabolically compromised horses (*p* ≥ 0.05; Figure 3).

### 3.3. Hemolysis parameters and distance ran

**Figure 4:**
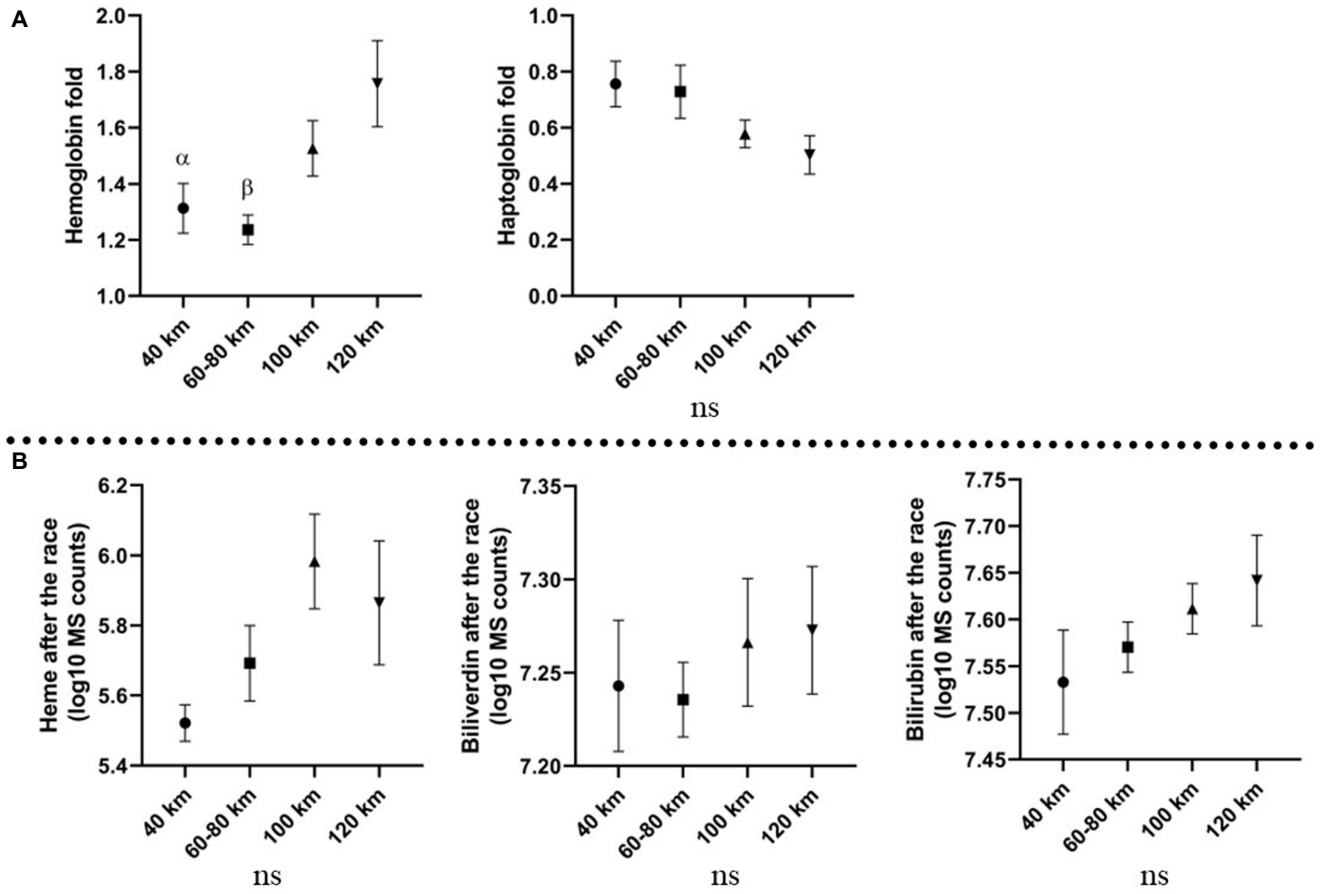
Association between distance ran and investigated molecules; **(A)** fold changes of the molecules analyzed with ELISA on different distance; **(B)** levels of molecules analyzed with metabolomics platform after the race on different distances, expressed in log10 MS counts; α: *p* ≤ 0.05, β: *p* ≤ 0.001 vs. 120 km; Mean values and standard errors of the mean are displayed.

Horses were divided into four groups, based on the distance ran (40, 60–80, 100, and 120 km). A significant association was found between plasma color and distance ran (Figure 2B), suggesting enhanced hemolysis under longer distances. A similar trend was observed by Hb fold, which was higher on longer distances (100–120 km) than the shorter distances (40–80 km). Heme, biliverdin and bilirubin produced no significant changes but displayed similar tendency, with higher levels associated with the longer distances than shorter distances. Hp decrease was more prominent on the longer distances than on the short distances. The described association between molecules and distance ran are displayed in Figure 4.

### 3.4. Hemolysis parameters and average speed

**Figure 5:**
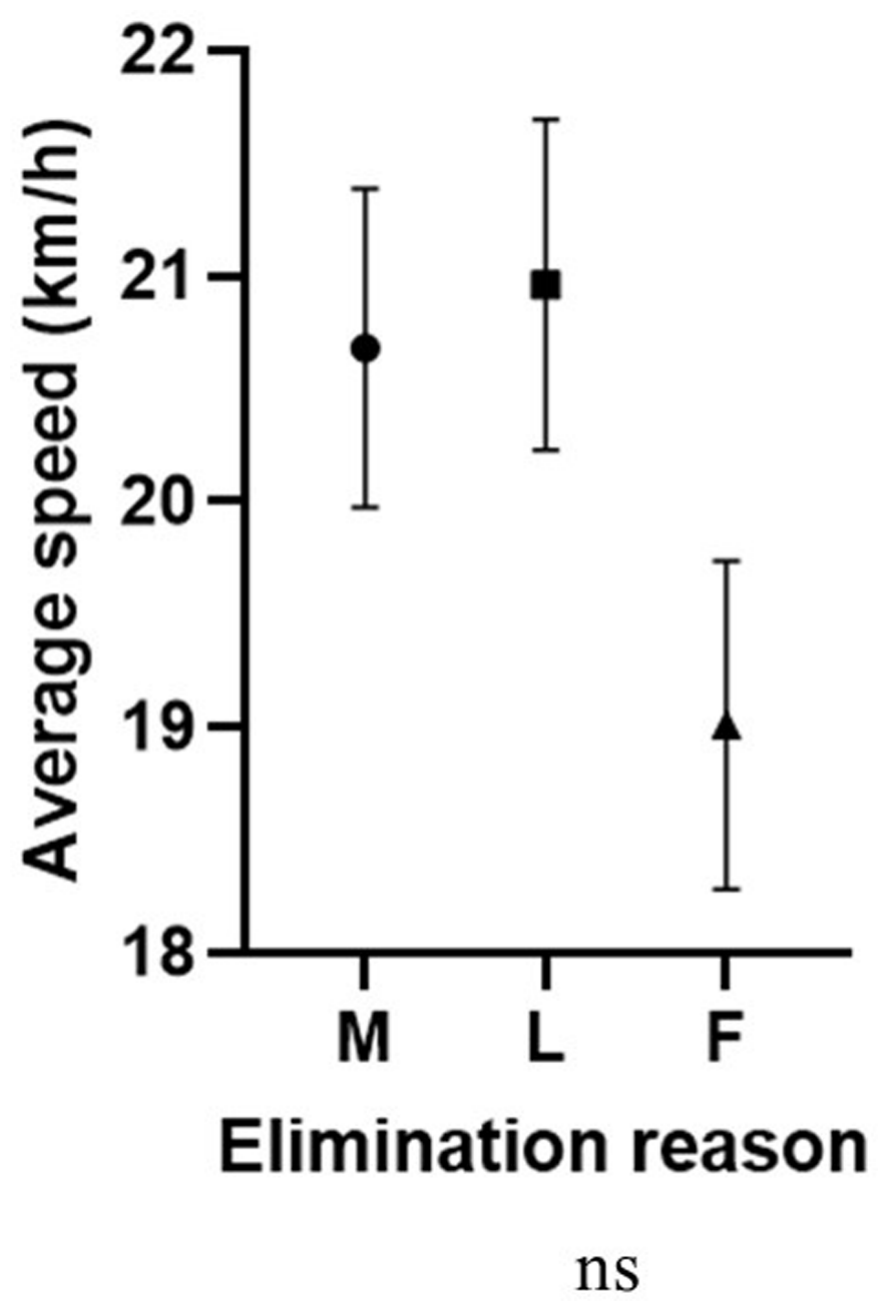
Association between average speed (km/h) and elimination reason (ns). M, metabolic eliminations; L, lameness eliminations; F, finishers.

Average speed was significantly associated with plasma color change, with higher values in more hemolyzed samples (higher plasma color change grade), than in non-hemolyzed specimens (Figure 2C). Interestingly, the comparison between average speed and the elimination reason showed that in the group of finishers, the average speed was lower than in horses eliminated for metabolic or lameness reasons (Figure 5).

### 3.5. Hemolysis parameters and heart rate

An interesting association was found between heart rate after the race and plasma color change assessed macroscopically. The trend displayed higher heart rate (HR) values in horses from which plasma samples were more hemolyzed (Figure 2D). Average HR for horses with no change in plasma color (grade 0) was 50.3 bpm (beats per minute), while horses with plasma color change grades 1, 2, and 3 presented heart rates of 61.6, 63.4, and 63.8 bpm, respectively.

## 4. Discussion

The present study has successfully displayed performance correlations with the level of molecules involved in hemolysis on endurance racing horses combining conventional methods of analysis with metabolomics, confirmed by the increase of Hb levels and all products of its degradation. Additionally, macroscopical changes in plasma color indicated that erythrolysis occurred during the race.

The association between all investigated molecules, as well as plasma color change, and distance ran by the horse validates that the severity of hemolysis is interconnected with the intensity of exercise. This effect could be linked with the increase number of foot strike leading to erythrocyte rupture. Additionally, it supports the fact that plasma color change is correlated with the average speed. Ultimately, longer distances and higher average speed are likely to increase the prominence of exercise-induced hemolysis. In the light of the presented results, riders and trainers should avoid reaching a certain speed/distance that is not adequate to the horse capabilities.

The high Hb and its degradation metabolites levels, as well as the lowest Hp levels in the group of horses eliminated for metabolic reasons may suggest a relationship between high levels of hemolysis and metabolic failure. Those metabolic disturbances, such as metabolic acidosis, dehydration or increased temperature, affect the fragility of the erythrocytes, making them prone to disruption. As previously described, a high level of free-Hb results in an increased consumption of NO leading to smooth muscle dystonia, vasculopathy, and endothelial dysfunction (28), and will consequently derive into colic, decreased peristalsis, respiratory distress, or cardiovascular impairment, which are common causes for elimination in endurance races. The current fact has been previously studied where researchers described the causes of colic in 36 endurance horses after the race, and in 56% of cases the diagnosis was uncertain or due to ileus (46), suggesting that a link could exist between gastrointestinal dystonias and hemolysis.

In humans, free heme, arising during hemolysis, has a toxic effect on the cells and organs, including kidneys, liver, nervous system, and cardiac tissue, acting as a pro-inflammatory molecule (47). In the present study, heme was the highest in horses eliminated for metabolic reasons, which leads to the conclusion that toxic effects of free heme may affect horses during endurance races, leading to metabolic impairment. Bilirubin displayed the highest levels in metabolically compromised horses, and is known to have neurotoxic properties (48). Given the fact that in this study, the group of horses eliminated for metabolic reasons had the highest levels of hemolysis parameters, contribution of exercise-induced hemolysis in the pathological processes should be kept in mind by the treating veterinarian when dealing with metabolically compromised horses after an endurance race.

The above described associations should encourage endurance participants to minimize the metabolic disturbances during the race by maintaining electrolytes balance, avoiding excessive heat or lowering lactates production with available supplements and decreasing lactic acid build-up. In the light of a previous published study by Bazzano and collaborators, describing the protective effect of polyunsaturated fatty acid on the osmosis of erythrocytes, trainers should also be considered, supplementing the animals with those specific lipids (34).

The occurrence of excessive hemolysis could be used by the equine practitioner, as a potential pre-indicator tool for the onset of metabolic diseases. Therefore, a pro-active treatment could be implemented to avoid catastrophic consequences, such as laminitis or colic. Notably, this study proves that change in plasma color is caused by intravascular hemolysis, and not by the hemoconcentration, believed to be linked to dehydration, which is of clinical importance. Thus, macroscopical plasma color change, although subjective, may be a helpful tool for immediate assessment of hemolysis severity for field practitioners.

It was shown that increased levels of Hb and heme resulting from intravascular hemolysis may induce platelet activation and thrombosis. Moreover, cytokines like IL-6, INF-γ, or IL-9, present in erythrocytes have pro-thrombotic properties (31, 49). A study performed on ponies confirmed that microvascular thrombosis exists during acute onset of laminitis. These data suggested that systemic coagulopathy might precede the development of the disease (50).

Laminitis as a consequence of endurance racing in horses with signs of exhaustion has been reported (51). The pathophysiology of endurance-related laminitis may be explained by repetitive foot strikes traumatizing the foot capillaries and the foot itself, leading to inflammation. Moreover, thrombosis, together with vasoconstriction and poor peripheral blood flow caused by elevated levels of catecholamines, cytokines, and cortisol, may contribute to this pathophysiological process (37). A recent study displayed that 21.1% of all orthopedic injuries originates from the foot (52). In some horses, signs of laminitis can be seen within hours of elimination/finishing the race, but other horses may need days to develop clinical manifestation (53). Therefore, measures should be taken in order to lower the risk of laminitis, by decreasing the hemolysis development with supplements, as previously described, but also by taking into consideration the surface type and specific adapted shoeing as described in the human field (20). Studies in this area are lacking and should be considered, to avoid this serious condition, often seen in endurance horses after a race.

According to the presented results obtained with colorimetric assays, the Hb increase was much less prominent in comparison with previous studies performed on standardbred race horses or on human athletes (140% increase in this study vs. 411% increase in standardbreds) (15, 33). This may be due to the time lapsed before finishing the exercise and blood collection. In the study performed on standardbred horses, the blood was collected within 5 min after the race. In the present study, the time between finishing the race and blood collection was longer (up to 30 min as the horse was undergoing a veterinary check). This fact may also lead to the conclusion that short, maximum intensity exercise like harness racing causes more severe hemolysis than long, moderate intensity racing, such as endurance events.

## 5. Conclusion

The current study provides an in-depth characterization of exercise-induced hemolysis by demonstrating the association between the severity of hemolysis, analyzed by conventional and metabolomics methods and the intensity of exercise, confirming the importance of appropriate adjustment of speed and distance to the horse capability. Moreover, the obtained results displayed the relationship between hemolysis and metabolic compromise. Complex analysis, including osmotic fragility of the erythrocytes, red blood cells count, cytokine analysis, clotting parameters, as well as objective assessment of plasma color change, should be performed in the future. Nevertheless, the presented study serves as prove of the occurrence of exercise-induced hemolysis in endurance horses, and encourages the endurance race industry stakeholders to consider the risk of hemolysis and its effects on the horses health and welfare.

## Data availability statement

The original contributions presented in the study are included in the article/supplementary material, further inquiries can be directed to the corresponding author.

## Ethics statement

The animal study was reviewed and approved by Institutional Animal Care and Use Committee (IACUC) of Weill Cornell Medicine, Qatar, under the number WCMQ-2018-003. Written informed consent was obtained from the owners for the participation of their animals in this study.

## Author contributions

PP: conceptualization, methodology, writing, and editing. AH and TV: conceptualization, methodology, writing, reviewing, and editing. EA-D and SJ: methodology, reviewing, and editing. SF: methodology and reviewing. KS: conceptualization, reviewing, and editing. All authors contributed to the article and approved the submitted version.

## Funding

The authors declare that this study received funding from he Intramural Grant Program of The Equine Veterinary Medical Center (EVMC), Doha, Qatar, grant number RG18_TV1. The funder was not involved in the study design, collection, analysis, interpretation of data, the writing of this article or the decision to submit it for publication.

## Conflict of interest

PP, EA-D, SJ, and TV were employed by Qatar Foundation. SF was employed by Al Shaqab.

The remaining authors declare that the research was conducted in the absence of any commercial or financial relationships that could be construed as a potential conflict of interest.

## Publisher’s note

All claims expressed in this article are solely those of the authors and do not necessarily represent those of their affiliated organizations, or those of the publisher, the editors and the reviewers. Any product that may be evaluated in this article, or claim that may be made by its manufacturer, is not guaranteed or endorsed by the publisher.
